# Are biologically meaningful effect sizes a factor in study design? A systematic review of translational chronic variable stress studies

**DOI:** 10.1113/EP092884

**Published:** 2025-07-09

**Authors:** Crispin Jordan, Nicola Romanò, John Menzies

**Affiliations:** ^1^ Centre for Discovery Brain Sciences, Edinburgh Medical School: Biomedical Sciences University of Edinburgh Edinburgh UK; ^2^ University of Edinburgh–Zhejiang University Joint Institute, Zhejiang University School of Medicine Zhejiang University Haining China

**Keywords:** chronic stress, effect size, experimental design

## Abstract

The design of in vivo studies using laboratory animals is normally guided by the 3Rs: Replacement, Reduction and Refinement. The concept of Reduction is particularly important in the context of estimating sample size; the selected sample size should allow the detection of a predetermined effect size using appropriate statistical tests, but not at the expense of using too many animals. To explore this, we conducted a systematic review of animal studies using chronic variable stress to ask whether the authors used a biologically meaningful effect size to determine the sample size. Only one article in our sample of 385 reported doing this, and most articles did not report a justification for the sample size used. Determining a biologically meaningful effect size is not always straightforward, but all appropriately powered studies based on a biologically meaningful effect size are useful, including studies where the data do not support the hypothesis. Accordingly, we believe the use of biologically meaningful effect sizes is central to decisions about study design and interpretation, and we discuss reasons and ways to promote its use.

## Abstract (highlights)

- **What is the topic of this review?**
  This systematic review explores whether biologically meaningful effect sizes are used in sample size calculations in translational studies of chronic variable stress.
- **What advances does it highlight?**
  We find that only a few studies give a clear justification for the sample size used, and in those that do, only a very small fraction used a biologically meaningful effect size as part of the sample size calculation. We discuss challenges around identifying a biologically meaningful effect size and ways to overcome them.

## 1 INTRODUCTION

The 3Rs (Replacement, Reduction and Refinement) are a framework for research using animals. Reduction refers to a ‘reduction in the numbers of animals used to obtain information of a given amount and precision’ (Russell & Burch, 1959), and a key Reduction‐related component of study design is selection of an appropriate sample size, i.e., using an appropriate number of animals to detect a particular effect size given a particular experimental and statistical design (Landis et al., 2012). Reporting a justified calculation of sample size ideally involves defining the variable to be measured and defining the magnitude of the effect size that is considered to be biologically meaningful, given the variance and correlation structures (e.g., repeated measures) in a study. In general, researchers are interested in making observations and finding interventions that are important biologically. A biological meaningful effect size is normally defined as an effect size that, if obtained, warrants further study. Accordingly, the use of a biologically meaningful effect size in sample size calculation could be viewed as one element of a rigorous approach to study design (Wilson et al., 2023).

The concept of a biological meaningful effect size is emphasized by the National Centre for the Replacement, Refinement and Reduction of Animals in Research and specified in the ARRIVE 2.0 guidelines (Sert et al., 2020). However, reporting the use of biological meaningful effect sizes to calculate sample size does not seem to be widespread. For example, after the introduction of a reporting checklist in a high‐profile neuroscience journal, only 7% of articles reported using a power calculation to determine the sample size (Carter et al., 2017). Some studies have noted small improvements in reporting of sample size (Hair et al., 2018; Han et al., 2017), but similarly low levels of reporting of sample size calculation are documented in systematic reviews of a range of translational studies (Baker et al., 2014; Bara & Joffe, 2014; Berrio et al., 2024; Brent et al., 2021; Carneiro et al., 2018; Chitolina et al., 2023; Codorniu et al., 2018; Eastwood et al., 2015; Faggion Jr et al., 2011; Farrell et al., 2014; Gallas‐Lopes et al., 2023; Hesen et al., 2017; Kilkenny et al., 2009; Kousholt et al., 2022; Moja et al., 2014; Rooke et al., 2011) and in an online ‘living review’ of transgenic animal models of Alzheimer's disease ( https:camarades.shinyapps.io/LivingEvidence_AD/).

Basing a sample size calculation on a biologically meaningful effect size is in line with the 3Rs principles, but only a minority of studies report how these principles inform study design (Kousholt et al., 2022). The relatively low prevalence of reporting a sample size calculation might be influenced by the fact it can be challenging to define a biologically meaningful effect size. Doing so requires an understanding the biological system being studied, the appropriateness of the methods used and the interventions tested. To explore this, we decided to document sample size calculations in studies using chronic variable stress (CVS) procedures, also called chronic unpredictable stress or chronic mild stress (Willner, 2017). We chose to study CVS procedures because they are likely to cause some degree of suffering to the animals involved, hence taking a rigorous and ethical approach to study design is of particular importance. Animals involved in a CVS study with too small a sample size will have experienced some degree of suffering in order to generate potentially unreliable data (Button et al., 2013; Wilson et al., 2023). Likewise, some of the animals involved in a study with too large a sample size need not be involved in order for the researchers to obtain reliable data (Jennions & Møller, 2003). Both situations are arguably unethical (Landis et al., 2012; Strech & Dirnagl, 2019). We conducted a systematic review to explore whether translational research articles reporting the use of CVS procedures included sample size calculations and used a biologically meaningful effect size in this calculation.

## 2 MATERIALS AND METHODS

**FIGURE 1:**
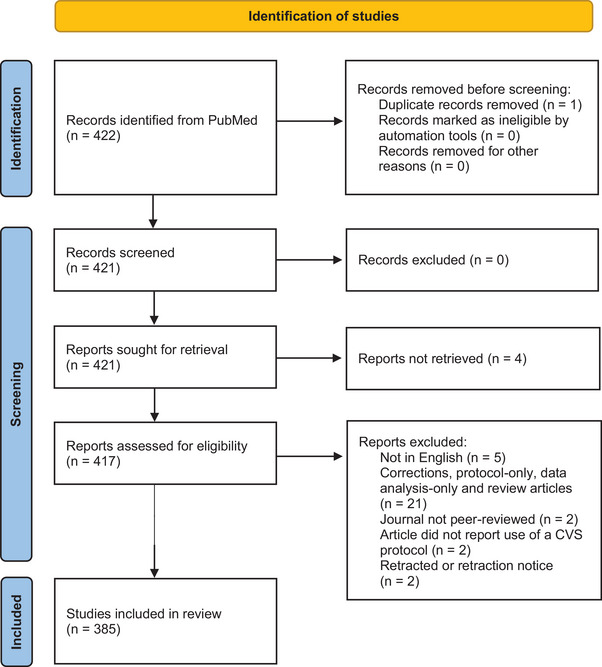
Identification of studies following Preferred Reporting Items for Systematic reviews and Meta‐Analyses (PRISMA) guidelines (Page et al., 2021). Abbreviation: CVS, chronic variable stress.

We searched PubMed for the terms ‘chronic variable stress’ and ‘chronic unpredictable stress’ in the Title/Abstract field. The search was done on 31 October 2023 and included all articles published from November 2018 (i.e., a 5 year period). We followed Preferred Reporting Items for Systematic reviews and Meta‐Analyses (PRISMA) guidelines on selecting and screening articles and on applying inclusion/exclusion criteria (Page et al., 2021). Details are shown in Figure 1.

To explore whether and how sample size had been calculated and whether effect size was a factor in this calculation, we searched the online text of each article at the journal website and all associated Supporting Information files for all instances of the words: ‘sample’, ‘effect’, ‘group’, ‘size’ and ‘power’. Journal websites were accessed between 31 October 2023 and 22 April 2024. A single researcher recorded each instance and documented the relevant text from the article. All comments on sample size and effect size were subject to thematic analysis (Braun & Clarke, 2012) to determine whether the full‐text article contained a justification for the number of experimental units used in the experimental groups, whether this justification was based on a power calculation and, if it was, what was the source of the effect size used in that power calculation. We also systematically documented other key indicators of study quality: the use of exclusion criteria, use of random allocation to experimental groups, and experimenter blinding to groups (Landis et al., 2012). To do this, the online text of each article was searched using the terms ‘exclu’, ‘inclu’, ‘criteri’, ‘random’ and ‘blind’. The researcher read all sections containing these flagged terms to determine their context. Relevant text was extracted and classified as shown in Supporting Data 2.

## 3 RESULTS

Most articles included in our review reported studies with commonly used laboratory rodents: 199 articles (52% of the total) used mice, 160 articles (42%) used rats, and five articles used both rats and mice. Sixteen articles used zebrafish, two used *Drosophila melanogaster*, one used California deermice (*Peromyscus californicus*), one used Japanese quail (*Coturnix japonica*), and one used cynomolgus monkeys (*Macaca fascicularis*).

**FIGURE 2:**
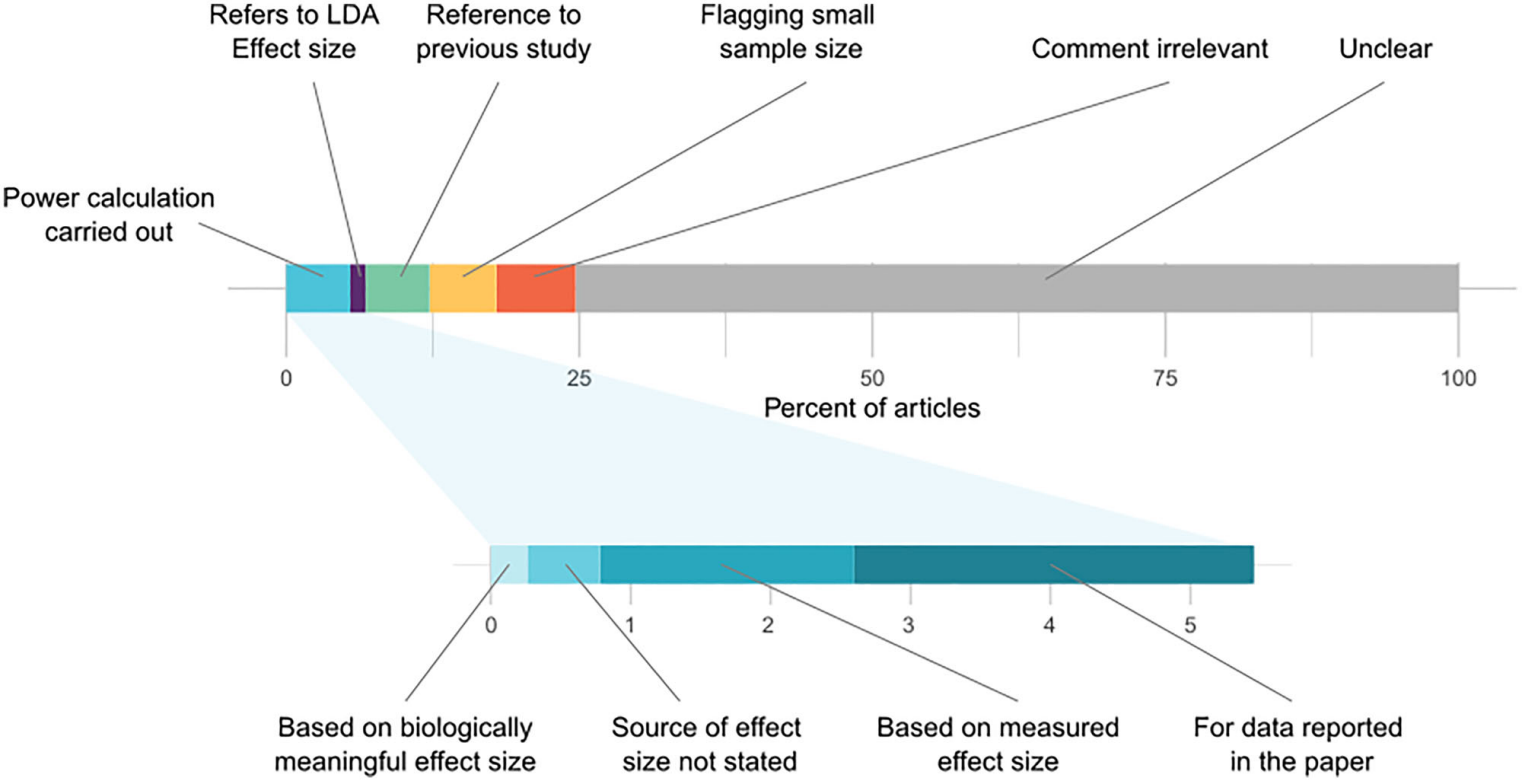
Quantitative summary of a thematic analysis of sample size reporting in the included articles. LDA is linear discriminant analysis.

Only one article (0.3% of the total) reported using a biologically meaningful effect size in order to determine a sample size (Ünal et al., 2023; Figure 2; Supporting Data 1). The corresponding author confirmed that the sample size calculation was based on a biologically meaningful effect size (Prof Özlem Özmen, personal communication, 19 November 2023). Of the remaining 384 articles, 298 articles (77%) made no explicit comment on either the sample size used and/or effect sizes. Of the articles that did mention sample size or effect size, 21 articles (6% of the total) stated that the sample size was based on data in previous literature or the authors’ own previous work. However, only one of these articles specified how previous work informed the determination of sample size. Seven articles (2%) stated that the sample size was calculated with a power calculation using data from a specific previous study. Two articles stated that a power calculation was used to determine the sample size but did not state the source of the effect size used. Two articles reported using Mead's resource equation (Mead, 1988) to calculate the sample size. Comments on sample size in 26 articles (7%) were general in nature rather than explanatory in terms of calculating the sample size (for example: ‘all efforts were made to reduce sample size and minimise animal suffering’). Comments made in 22 articles (6%) were to flag that the sample size used in the study might have been too small. Eleven articles (3%) carried out a *post hoc* power analysis. However, it is generally accepted that, if the *p*‐value is reported, calculating the power *post hoc* does not provide additional information (Hoenig & Heisey, 2001). Five articles contained the phrase ‘effect size’ in the context of linear discriminant analysis of effect size (Klaus, 2012).

We also systematically documented other key indicators of study quality. Details are given in the Supporting Text, but in summary, 19% of the included articles reported using exclusion criteria, 46% reported that experimental units were allocated randomly to the control and CVS groups, and 35% reported that experimenters were blinded during the collection and/or analysis of data.

## 4 DISCUSSION

We believe that most researchers want their work to matter, both in the sense of having a positive impact with research and public/patient communities and in the sense of mattering biologically and/or clinically. However, we would argue that almost any result is of limited utility (regardless of whether it is statistically significant) if it is not embedded in a sense of what a biologically meaningful effect size is in that context. Only 1 article of the 385 included in our review reported using a biologically meaningful effect size to calculate a sample size, and only a further 7 articles described using a power calculation to justify the sample size used. Our observations are in line with other analyses of in vivo animal studies, where a median of only 2% of articles state how sample size was calculated (Baker et al., 2014; Bara & Joffe, 2014; Brent et al., 2021; Carneiro et al., 2018; Chitolina et al., 2023; Codorniu et al., 2018; Eastwood et al., 2015; Faggion Jr et al., 2011; Farrell et al., 2014; Gallas‐Lopes et al., 2023; Hesen et al., 2017; Kilkenny et al., 2009; Kousholt et al., 2022; Rooke et al., 2011). Significant efforts have been made to improve the experimental and statistical design of translational studies, particularly the ARRIVE guidelines (Sert et al., 2020) and the adoption of reporting checklists by journals. These efforts have had a positive impact in many important respects, but a study on the impact of requiring authors to comply with ARRIVE guidelines showed a limited effect on real compliance (Hair et al., 2018), and calls to improve the reporting of sample size calculations continue (Bespalov et al., 2021; Macleod & Howells, 2016; Piper et al., 2023).

We did not calculate *post hoc* whether the samples sizes used in the articles in our study were appropriate in terms of detecting the observed effect size. However, we previously noted that the median sample size reported in a subset of the articles used for the present study ‐ a subset featuring the sucrose preference test (SPT) and forced swim test (FST) as a means of evaluating the effects of CVS ‐ was 10 animals per group (Romanò & Menzies, 2025). This is in line with what has been described as an ‘intuition’ of adequate sample size (Smalheiser et al., 2021). It is not clear whether this intuition is based predominantly on statistical or biological understanding, on practical considerations and/or on tradition, but published studies are frequently underpowered (Button et al., 2013), indicating that this intuition is sometimes incorrect. Indeed, a comparison between two independent groups with a sample size of 10 (using a two‐tailed *t*‐test, α = 0.05, power = 0.8) allows the detection of an effect size of 1.3 (Cohen's *d*). This is close to the median effect size of 1.6 we observed in our study of post‐CVS SPT and FST effect size data extracted from a subset of the articles included in the present study. At any rate, the use of incorrectly overestimated effect sizes to calculate a sample size, combined with a publication bias for results with a *p*‐value of <0.05, might lead to a biased view of what a suitable effect size is and inflate the frequency of published false‐positive results (Ioannidis, 2005).

The selection of a sample size also relates to the 3Rs. In a meta‐analysis of statistical power in neuroscience research, Button et al. (2013) noted that the average sample sizes used in a common behavioural test were capable of detecting only a relatively large effect size (Cohen's *d* = 1.2), but that the likely true, smaller effect size (Cohen's *d* = 0.5) needed a sample size of 134 animals to achieve 80% power. They note that the total number of animals used across all the studies that were included in their meta‐analysis was more than three times the number needed to detect this small effect size in a single study designed in order to detect that effect size. In other words, the cumulative use of animals across a series of independent but unreliably powered studies might result in the use of more animals than a single study appropriately designed to detect a small effect size.

To be clear, we do not insist that a power calculation must always be used to determine sample size. For example, power analysis becomes less crucial if the focus of researchers is on estimating an effect size and its associated uncertainty rather than on *p*‐values [for example, 95% confidence intervals (CI) for the effect size]; in particular, the common (but arguably misplaced) focus on whether the *p*‐value is <0.05 (Amrhein et al., 2019; Wasserstein et al., 2019). Even then, however, methods of power analysis exist to design experiments to estimate effect size by, for example, using simulations and replacing *p *< 0.05 with a desirably small 95% CI as the criterion for a ‘successful’ experiment (Colegrave & Ruxton, 2020). Furthermore, power analysis might not be required for purely exploratory work, but this requires authors to articulate which components of reported research are ‘exploratory’ and which are ‘confirmatory’ (Wagenmakers et al., 2012). Importantly, exploratory research can help researchers to select a biologically meaningful effect size, accounting for both point estimates and confidence limits (Amrhein et al., 2019). Care is needed when using (exploratory) research to identify a biologically meaningful effect size. For example, effect sizes in published but underpowered studies are likely to be inflated (Colquhoun, 2014), and pilot studies, by their nature, provide unreliable estimates of effect sizes.

These issues highlight the need for all studies to report effect sizes along with appropriate measures of uncertainty (standard errors or 95% CI). By reporting effect sizes, authors: (1) draw the attention of the reader to the biological (un)importance of a reported effect, which adds understanding; and (2) provide crucial information to either conduct meta‐analyses or design follow‐up experiments. Importantly though, there is arguably no shared conception of what constitutes a small, medium or large effect size when expressed as, for example, Cohen's *d*. A Cohen's *d* value of 0.5 might be considered a ‘small effect’ in one biological context but a ‘large effect’ in another (Brydges, 2019). This makes it difficult for researchers to determine whether a somewhat abstract Cohen's *d* value (or a similar measure of effect size) constitutes a meaningful effect size in their context. To improve interpretation, we encourage authors also to report effect sizes on the biological scale at which an experiment occurred; e.g., reporting the time spent immobile in the FST in seconds, or the reduction in sucrose preference (in addition to absolute volumes consumed) in the SPT (Romanò & Menzies, 2025). Reporting effect sizes this way might be much more intuitive to readers than a more abstract expression of effect size.

So far, we have focused on an argument for identifying and using the smallest effect size of biological interest as a component of study design. However, knowing what is biologically meaningful is also crucial when interpreting results. For example, obtaining a small *p*‐value for an experimental treatment provides a researcher with one line of evidence that the treatment has an effect. However, if the researcher is unsure whether this effect is large enough to be biologically relevant, they do not have a way to judge whether this statistically significant result is meaningful. Regardless of the *p*‐value obtained, meaningful interpretation can occur only in the context of a sense of the minimal biologically important effect size (Amrhein et al., 2019; Wasserstein et al., 2019). To give a concrete example, in a clinical trial exploring the impact of exercise on physical and mental health in patients with coronary artery disease, the authors identified statistically significant effects of treatments on blood pressure (systolic blood pressure, 122 ± 14 vs. 126 ± 14 mmHg; *p* = 0.043), but concluded that the changes were unlikely to be clinically meaningful because participants remained normotensive (Terada et al., 2022); independent research indicates that changes of 5–10 mmHg (preferably closer to 10) constitute a clinically meaningful change in systolic blood pressure (Kandzari et al., 2022). In our view, this emphasis on meaningfulness was an appropriate and useful conclusion.

Importantly, the exact value of a biologically meaningful effect size may not be fixed. For example, new insights might change our view of what a biologically relevant effect size is, or the same effect size might be biologically relevant in one context and irrelevant in another (although the data might have the same level of statistical significance in both cases). However, if we want to encourage biological meaningfulness as a basis for these calculations, we are faced with a challenge. When writing about biological meaningfulness, Johnson et al. (2015) note that concerns are frequently expressed as a question: How can I power my study to detect an unknown effect size? They reply that “a study should be powered to detect not the actual effect (which cannot anyway be known before collecting the data) but the smallest effect … that, in the judgement of the researcher, is worth detecting”. But how should a researcher decide what is, in fact, worth detecting? The ability to make this decision will probably come from an understanding of the biological system, which, in turn, normally derives from good‐quality data scrutinized and interpreted by experts in the field. For instance, the clinical trial by Terada et al. (2022) benefitted from independent research that specifically aimed to determine minimally important effect sizes (Wise & Brown, 2005). When such data do not exist, research to determine important effect sizes becomes essential.

This research might include theoretical studies. One way forwards is to use mathematical models of biological processes. These models could be used to predict the effect size of the proposed intervention on a specific output of a biological system in the biomedical context, including behaviours (Hales et al., 2017) and physiological processes (Hassan et al., 2023). Other areas of biology, particularly ecology and evolution, have rich and ongoing histories of using mathematical models to understand biological processes. Consider an example in the field of evolutionary genetics, where a researcher might wish to study the potential of a species to adapt to a local environment. Mathematical models predict that a population of a given species might evolve adaptations to a local habitat or region at the expense of being maladapted to another region or habitat when the strength of natural selection is greater than the rate of immigration between the regions (Levene, 1953; Yeaman & Otto, 2011). Therefore, if the researcher has estimates of immigration rates, these estimates can be used as a minimal effect size to design experiments on natural selection.

Our study has a number of limitations. Firstly, we did not preregister our protocol design. Secondly, by setting strict inclusion criteria, we might not have captured all relevant articles. Setting these criteria is intended to reduce selection bias, but translational procedures that impose stressors over long periods are known by a variety of names (Willner, 2017), not only by our search terms, hence the included articles represent a sample of all relevant studies published in that time frame. We used specific keywords to search articles for a report of whether a biologically meaningful effect size was used as part of the justification for the sample size used. This approach is not validated and might result in a type 2 error (i.e., an underestimate of the number of articles that did use a biologically meaningful effect size). Screening, article inclusion/exclusion, data collection and thematic analyses were done manually by a single researcher, and this might introduce biases (Mackieson et al., 2019; Page et al., 2021; Waffenschmidt et al., 2019)

In conclusion, we found that only a few CVS studies used a power analysis to determine sample sizes, and even fewer used biologically informed effect sizes in their sample size calculations. This raises both scientific and ethical concerns. Scientifically, and in line with current advice (Sert et al., 2020), we support the routine use of an estimation of adequate sample size that is based on meaningful biological effects. This approach might improve the quality and reproducibility of study design and contribute positively to the critical interpretation of data. The meaningful interpretation of any result, regardless of the *p*‐value obtained, is highly challenging without a sense of what a biologically meaningful effect size is likely to be. Accordingly, if there is limited understanding or agreement of what a biologically meaningful effect size is in a given context, research to articulate this should be a priority. In terms of ethical considerations, Reduction is normally regarded as an ethically sensitive approach to study design, but care needs to be taken in at least two respects. Firstly, using a sample size that is too small brings the potential risk of unreliable data ‐ specifically, higher probabilities of type 2 errors generally, and type 1 errors among published results owing to publication bias for *p* < 0.05; Ioannidis, 2005) ‐ and holds the potential to lead researchers down unproductive lines of scientific inquiry. All research entails an investment of time and resources, and because of this, poor study design is wasteful on many levels. Secondly, Reduction might not be appropriate if it increases the level of individual suffering for the animals taking part in the experiment (Tannenbaum & Bennett, 2015). Researchers should consider the difficult ethical balance between minimizing the level of suffering experienced by an individual animal and minimizing the sum of suffering experienced by all the animals involved in a study. In other words, the ethical question of whether it is better for a small number of animals to experience a high level of suffering or for a larger number of animals to experience a more moderate level of suffering. Given that an individual animal is likely to be very highly aware of the suffering it itself experiences, but much less aware (or even entirely unaware) of how the sum total of suffering is distributed between itself and the other animals in the study, the latter is arguably preferable.

## AUTHOR CONTRIBUTIONS

Crispin Jordan: Conceptualization, methodology and writing – review & editing. Nicola Romanò: Funding acquisition, methodology and writing – review & editing. John Menzies: Conceptualization, investigation, methodology, formal analysis, writing – original draft preparation and project administration. All authors approved the final version of the manuscript and agree to be accountable for all aspects of the work in ensuring that questions related to the accuracy or integrity of any part of the work are appropriately investigated and resolved. All persons designated as authors qualify for authorship, and all those who qualify for authorship are listed.

## CONFLICT OF INTEREST

The authors declare no conflicts of interest.

## Supporting information

Supplementary Materials.

Supplementary Materials.

Supplementary Materials.

## Data Availability

All data collected in this study are provided as Supplementary Data.

## References

[eph13923-bib-0001] Amrhein, V. , Greenland, S. , & McShane, B. (2019). Scientists rise up against statistical significance. Nature, 567(7748), 305–307. 30894741 10.1038/d41586-019-00857-9

[eph13923-bib-0002] Baker, D. , Lidster, K. , Sottomayor, A. , & Amor, S. (2014). Two years later: Journals are not yet enforcing the ARRIVE guidelines on reporting standards for pre‐clinical animal studies. PLoS Biology, 12(1), e1001756. 24409096 10.1371/journal.pbio.1001756 PMC3883646

[eph13923-bib-0003] Bara, M. , & Joffe, A. R. (2014). The ethical dimension in published animal research in critical care: The public face of science. Critical Care, 18(1), R15. 24423201 10.1186/cc13694 PMC4056799

[eph13923-bib-0004] Berrio, J. P. , Hestehave, S. , & Kalliokoski, O. (2024). Reliability of sucrose preference testing following short or no food and water deprivation‐a Systematic Review and Meta‐Analysis of rat models of chronic unpredictable stress. Translational Psychiatry, 14(1), 39. 38242881 10.1038/s41398-024-02742-0 PMC10799054

[eph13923-bib-0005] Bespalov, A. , Bernard, R. , Gilis, A. , Gerlach, B. , Guillén, J. , Castagné, V. , Lefevre, I. A. , Ducrey, F. , Monk, L. , Bongiovanni, S. , Altevogt, B. , Arroyo‐Araujo, M. , Bikovski, L. , de Bruin, N. , Castaños‐Vélez, E. , Dityatev, A. , Emmerich, C. H. , Fares, R. , Ferland‐Beckham, C. , … Steckler, T. (2021). Introduction to the EQIPD quality system. eLife, 10, e63294. 34028353 10.7554/eLife.63294 PMC8184207

[eph13923-bib-0006] Braun, V. , & Clarke, V. (2012). Thematic analysis. APA handbook of research methods in psychology: Vol. 2. Research designs: Quantitative, qualitative, neuropsychological, and biological (pp. 57–71). American Psychological Association. 10.1037/13620-004

[eph13923-bib-0007] Brent, M. B. , Brüel, A. , & Thomsen, J. S. (2021). A systematic review of animal models of disuse‐induced bone loss. Calcified Tissue International, 108(5), 561–575. 33386477 10.1007/s00223-020-00799-9

[eph13923-bib-0008] Brydges, C. R. (2019). Effect size guidelines, sample size calculations, and statistical power in gerontology. Innovation in Aging, 3(4), igz036. 31528719 10.1093/geroni/igz036 PMC6736231

[eph13923-bib-0009] Button, K. S. , Ioannidis, J. P. A. , Mokrysz, C. , Nosek, B. A. , Flint, J. , Robinson, E. S. J. , & Munafò, M. R. (2013). Power failure: Why small sample size undermines the reliability of neuroscience. Nature Reviews Neuroscience, 14(5), Article 5. 10.1038/nrn3475 23571845

[eph13923-bib-0010] Carneiro, C. F. D. , Moulin, T. C. , Macleod, M. R. , & Amaral, O. B. (2018). Effect size and statistical power in the rodent fear conditioning literature—A systematic review. PLoS ONE, 13(4), e0196258. 29698451 10.1371/journal.pone.0196258 PMC5919667

[eph13923-bib-0011] Carter, A. , Tilling, K. , & Munafò, M. R. (2017). A systematic review of sample size and power in leading neuroscience journals (p. 217596). Advance online publication. 10.1101/217596

[eph13923-bib-0012] Chitolina, R. , Gallas‐Lopes, M. , Reis, C. G. , Benvenutti, R. , Stahlhofer‐Buss, T. , Calcagnotto, M. E. , Herrmann, A. P. , & Piato, A. (2023). Chemically‐induced epileptic seizures in zebrafish: A systematic review. Epilepsy Research, 197, 107236. 37801749 10.1016/j.eplepsyres.2023.107236

[eph13923-bib-0013] Codorniu, A. , Lemasle, L. , Legrand, M. , Blet, A. , Mebazaa, A. , & Gayat, E. (2018). Methods used to assess the performance of biomarkers for the diagnosis of acute kidney injury: A systematic review and meta‐analysis. Biomarkers, 23(8), 766–772. 29943660 10.1080/1354750X.2018.1493616

[eph13923-bib-0014] Colegrave, N. , & Ruxton, G. D. (2020). Power analysis: An introduction for the life sciences. Oxford University Press.

[eph13923-bib-0015] Colquhoun, D. (2014). An investigation of the false discovery rate and the misinterpretation of p‐values. Royal Society Open Science, 1(3), 140216. 26064558 10.1098/rsos.140216 PMC4448847

[eph13923-bib-0016] Eastwood, M. P. , Russo, F. M. , Toelen, J. , & Deprest, J. (2015). Medical interventions to reverse pulmonary hypoplasia in the animal model of congenital diaphragmatic hernia: A systematic review. Pediatric Pulmonology, 50(8), 820–838. 25994108 10.1002/ppul.23206

[eph13923-bib-0017] Faggion, Jr, C. M. , Giannakopoulos, N. N. , & Listl, S. (2011). Risk of bias of animal studies on regenerative procedures for periodontal and peri‐implant bone defects—a systematic review. Journal of Clinical Periodontology, 38(12), 1154–1160. 22092584 10.1111/j.1600-051X.2011.01783.x

[eph13923-bib-0018] Farrell, K. E. , Keely, S. , Graham, B. A. , Callister, R. , & Callister, R. J. (2014). A systematic review of the evidence for central nervous system plasticity in animal models of inflammatory‐mediated gastrointestinal pain. Inflammatory Bowel Diseases, 20(1), 176–195. 24284415 10.1097/01.MIB.0000437499.52922.b1

[eph13923-bib-0019] Gallas‐Lopes, M. , Bastos, L. M. , Benvenutti, R. , Panzenhagen, A. C. , Piato, A. , & Herrmann, A. P. (2023). Systematic review and meta‐analysis of 10 years of unpredictable chronic stress in zebrafish. Lab Animal, 52(10), Article 10. 10.1038/s41684-023-01239-5 37709998

[eph13923-bib-0020] Hair, K. , Macleod, M. , & Sena, E. . (2018). A randomised controlled trial of an Intervention to Improve Compliance with the ARRIVE guidelines (IICARus) (p. 370874). Advance online publication. 10.1101/370874 PMC6560728 31205756

[eph13923-bib-0021] Hales, C. A. , Houghton, C. J. , & Robinson, E. S. J. (2017). Behavioural and computational methods reveal differential effects for how delayed and rapid onset antidepressants effect decision making in rats. European Neuropsychopharmacology, 27(12), 1268–1280. 29100819 10.1016/j.euroneuro.2017.09.008 PMC5720479

[eph13923-bib-0022] Han, S. , Olonisakin, T. F. , Pribis, J. P. , Zupetic, J. , Yoon, J. H. , Holleran, K. M. , Jeong, K. , Shaikh, N. , Rubio, D. M. , & Lee, J. S. (2017). A checklist is associated with increased quality of reporting preclinical biomedical research: A systematic review. PLoS ONE, 12(9), e0183591. 28902887 10.1371/journal.pone.0183591 PMC5597130

[eph13923-bib-0023] Hassan, S. , El Baradey, H. , Madi, M. , Shebl, M. , Leng, G. , Lozic, M. , Ludwig, M. , Menzies, J. , & MacGregor, D. (2023). Measuring oxytocin release in response to gavage: Computational modelling and assay validation. Journal of Neuroendocrinology, 35(6), e13303. 37316906 10.1111/jne.13303 PMC10909523

[eph13923-bib-0024] Hesen, N. A. , Riksen, N. P. , Aalders, B. , Ritskes‐Hoitinga, M. , Messaoudi, S. E. , & Wever, K. E. (2017). A systematic review and meta‐analysis of the protective effects of metformin in experimental myocardial infarction. PLoS ONE, 12(8), e0183664. 28832637 10.1371/journal.pone.0183664 PMC5568412

[eph13923-bib-0025] Hoenig, J. M. , & Heisey, D. M. (2001). The abuse of power. The American Statistician, 55(1), 19–24.

[eph13923-bib-0026] Ioannidis, J. P. A. (2005). Why most published research findings are false. PLOS Medicine, 2(8), e124. 16060722 10.1371/journal.pmed.0020124 PMC1182327

[eph13923-bib-0027] Jennions, M. D. , & Møller, A. P. (2003). A survey of the statistical power of research in behavioral ecology and animal behavior. Behavioral Ecology, 14(3), 438–445.

[eph13923-bib-0028] Johnson, P. C. D. , Barry, S. J. E. , Ferguson, H. M. , & Müller, P. (2015). Power analysis for generalized linear mixed models in ecology and evolution. Methods in Ecology and Evolution, 6(2), 133–142. 25893088 10.1111/2041-210X.12306 PMC4394709

[eph13923-bib-0029] Kandzari, D. E. , Mahfoud, F. , Weber, M. A. , Townsend, R. , Parati, G. , Fisher, N. D. L. , Lobo, M. D. , Bloch, M. , Böhm, M. , Sharp, A. S. P. , Schmieder, R. E. , Azizi, M. , Schlaich, M. P. , Papademetriou, V. , Kirtane, A. J. , Daemen, J. , Pathak, A. , Ukena, C. , Lurz, P. , … Spitzer, E. (2022). Clinical trial design principles and outcomes definitions for device‐based therapies for hypertension: A consensus document from the hypertension academic research consortium. Circulation, 145(11), 847–863. 35286164 10.1161/CIRCULATIONAHA.121.057687 PMC8912966

[eph13923-bib-0030] Kilkenny, C. , Parsons, N. , Kadyszewski, E. , Festing, M. F. W. , Cuthill, I. C. , Fry, D. , Hutton, J. , & Altman, D. G. (2009). Survey of the quality of experimental design, statistical analysis and reporting of research using animals. PLoS ONE, 4(11), e7824. 19956596 10.1371/journal.pone.0007824 PMC2779358

[eph13923-bib-0031] Klaus, B. (2012). Effect size estimation and misclassification rate based variable selection in linear discriminant analysis. *arXiv*. 10.48550/arXiv.1205.6653

[eph13923-bib-0032] Kousholt, B. S. , Præstegaard, K. F. , Stone, J. C. , Thomsen, A. F. , Johansen, T. T. , Ritskes‐Hoitinga, M. , & Wegener, G. (2022). Reporting quality in preclinical animal experimental research in 2009 and 2018: A nationwide systematic investigation. PLoS ONE, 17(11), e0275962. 36327216 10.1371/journal.pone.0275962 PMC9632797

[eph13923-bib-0033] Landis, S. C. , Amara, S. G. , Asadullah, K. , Austin, C. P. , Blumenstein, R. , Bradley, E. W. , Crystal, R. G. , Darnell, R. B. , Ferrante, R. J. , Fillit, H. , Finkelstein, R. , Fisher, M. , Gendelman, H. E. , Golub, R. M. , Goudreau, J. L. , Gross, R. A. , Gubitz, A. K. , Hesterlee, S. E. , Howells, D. W. , … Silberberg, S. D. (2012). A call for transparent reporting to optimize the predictive value of preclinical research. Nature, 490(7419), Article 7419. 10.1038/nature11556 PMC3511845 23060188

[eph13923-bib-0034] Levene, H. (1953). Genetic equilibrium when more than one ecological niche is available. The American Naturalist, 87(836), 331–333.

[eph13923-bib-0035] Mackieson, P. , Shlonsky, A. , & Connolly, M. (2019). Increasing rigor and reducing bias in qualitative research: A document analysis of parliamentary debates using applied thematic analysis. Qualitative Social Work, 18(6), 965–980.

[eph13923-bib-0036] Macleod, M. , & Howells, D. (2016). Protocols for laboratory research. Evidence‐Based Preclinical Medicine, 3(2), e00021.

[eph13923-bib-0037] Mead, R. (1988). The design of experiments: Statistical principles for practical applications. Cambridge University Press. http://archive.org/details/designofexperime0000mead

[eph13923-bib-0038] Moja, L. , Pecoraro, V. , Ciccolallo, L. , Dall'Olmo, L. , Virgili, G. , & Garattini, S. (2014). Flaws in animal studies exploring statins and impact on meta‐analysis. European Journal of Clinical Investigation, 44(6), 597–612. 24665945 10.1111/eci.12264

[eph13923-bib-0039] Page, M. J. , Moher, D. , Bossuyt, P. M. , Boutron, I. , Hoffmann, T. C. , Mulrow, C. D. , Shamseer, L. , Tetzlaff, J. M. , Akl, E. A. , Brennan, S. E. , Chou, R. , Glanville, J. , Grimshaw, J. M. , Hróbjartsson, A. , Lalu, M. M. , Li, T. , Loder, E. W. , Mayo‐Wilson, E. , McDonald, S. , … McKenzie, J. E. (2021). PRISMA 2020 explanation and elaboration: Updated guidance and exemplars for reporting systematic reviews. British Medical Journal, 372, n160. 33781993 10.1136/bmj.n160 PMC8005925

[eph13923-bib-0040] Piper, S. K. , Zocholl, D. , Toelch, U. , Roehle, R. , Stroux, A. , Hoessler, J. , Zinke, A. , & Konietschke, F. (2023). Statistical review of animal trials—A guideline. Biometrical Journal, 65(2), 2200061. 10.1002/bimj.202200061 36071025

[eph13923-bib-0041] Romanò, N. , & Menzies, J. (2025). Rodent chronic variable stress procedures: A disjunction between stress entity and impact on behaviour. Journal of Neuroendocrinology, 37(9), e70051. 40497530 10.1111/jne.70051 PMC12404908

[eph13923-bib-0042] Rooke, E. D. M. , Vesterinen, H. M. , Sena, E. S. , Egan, K. J. , & Macleod, M. R. (2011). Dopamine agonists in animal models of Parkinson's disease: A systematic review and meta‐analysis. Parkinsonism & Related Disorders, 17(5), 313–320. 21376651 10.1016/j.parkreldis.2011.02.010

[eph13923-bib-0043] Russell, W. , & Burch, R. (1959). The principles of humane experimental technique. Methuen.

[eph13923-bib-0044] Sert, N. P. d. , Hurst, V. , Ahluwalia, A. , Alam, S. , Avey, M. T. , Baker, M. , Browne, W. J. , Clark, A. , Cuthill, I. C. , Dirnagl, U. , Emerson, M. , Garner, P. , Holgate, S. T. , Howells, D. W. , Karp, N. A. , Lazic, S. E. , Lidster, K. , MacCallum, C. J. , Macleod, M. , … Würbel, H. (2020). The ARRIVE guidelines 2.0: Updated guidelines for reporting animal research. PLoS Biology, 18(7), e3000410. 32663219 10.1371/journal.pbio.3000410 PMC7360023

[eph13923-bib-0045] Smalheiser, N. R. , Graetz, E. E. , Yu, Z. , & Wang, J. (2021). Effect size, sample size and power of forced swim test assays in mice: Guidelines for investigators to optimize reproducibility. PLoS ONE, 16(2), e0243668. 33626103 10.1371/journal.pone.0243668 PMC7904226

[eph13923-bib-0046] Strech, D. , & Dirnagl, U. (2019). 3Rs missing: Animal research without scientific value is unethical. BMJ Open Science, 3(1), bmjos–2018–000048. 10.1136/bmjos-2018-000048 PMC8647585 35047678

[eph13923-bib-0047] Tannenbaum, J. , & Bennett, B. T. (2015). Russell and Burch's 3Rs then and now: The need for clarity in definition and purpose. Journal of the American Association for Laboratory Animal Science: JAALAS, 54(2), 120–132. 25836957 PMC4382615

[eph13923-bib-0048] Terada, T. , Cotie, L. M. , Tulloch, H. , Mistura, M. , Vidal‐Almela, S. , O'Neill, C. D. , Reid, R. D. , Pipe, A. , & Reed, J. L. (2022). Sustained effects of different exercise modalities on physical and mental health in patients with coronary artery disease: A randomized clinical trial. Canadian Journal of Cardiology, 38(8), 1235–1243. 35961757 10.1016/j.cjca.2022.03.017

[eph13923-bib-0049] Ünal, G. Ö. , Erkılınç, G. , Öztürk, K. H. , Doguç, D. K. , & Özmen, Ö. (2023). The beneficial effects of vortioxetine on BDNF, CREB, S100B, β amyloid, and glutamate NR2b receptors in chronic unpredictable mild stress model of depression. Psychopharmacology, 240(12), 2499–2513. 37555927 10.1007/s00213-023-06445-0

[eph13923-bib-0050] Waffenschmidt, S. , Knelangen, M. , Sieben, W. , Bühn, S. , & Pieper, D. (2019). Single screening versus conventional double screening for study selection in systematic reviews: A methodological systematic review. BioMed Central Medical Research Methodology, 19(1), 132. 31253092 10.1186/s12874-019-0782-0 PMC6599339

[eph13923-bib-0051] Wagenmakers, E.‐J. , Wetzels, R. , Borsboom, D. , van der Maas, H. L. J. , & Kievit, R. A. (2012). An agenda for purely confirmatory research. Perspectives on Psychological Science, 7(6), 632–638. 26168122 10.1177/1745691612463078

[eph13923-bib-0052] Wasserstein, R. L. , Schirm, A. L. , & Lazar, N. A. (2019). Moving to a world beyond “p < 0.05”. The American Statistician, 73(Suppl 1), 1–19.

[eph13923-bib-0053] Willner, P. (2017). The chronic mild stress (CMS) model of depression: History, evaluation and usage. Neurobiology of Stress, 6, 78–93. 28229111 10.1016/j.ynstr.2016.08.002 PMC5314424

[eph13923-bib-0054] Wilson, E. , Ramage, F. J. , Wever, K. E. , Sena, E. S. , Macleod, M. R. , & Currie, G. L. (2023). Designing, conducting, and reporting reproducible animal experiments. Journal of Endocrinology, 258(1), e220330. 37074416 10.1530/JOE-22-0330 PMC10304908

[eph13923-bib-0055] Wise, R. A. , & Brown, C. D. (2005). Minimal clinically important differences in the six‐minute walk test and the incremental shuttle walking test. COPD: Journal of Chronic Obstructive Pulmonary Disease, 2(1), 125–129. 17136972 10.1081/copd-200050527

[eph13923-bib-0056] Yeaman, S. , & Otto, S. P. (2011). Establishment and maintenance of adaptive genetic divergence under migration, selection, and drift. Evolution; International Journal of Organic Evolution, 65(7), 2123–2129. 21729066 10.1111/j.1558-5646.2011.01277.x

