# Supplementary material for: Are biologically meaningful effect sizes a factor in study design? A systematic review of translational chronic variable stress studies
Source: Exp Physiol. 2025 Jul 9;111(2):349–56. doi: 10.1113/EP092884 (PMC12857510; doi:10.1113/EP092884)
Supplement: Supplementary file 1 — Supplementary Materials. [file EPH-111-349-s002.docx]

**Jordan, Romanò & Menzies**

**Are biologically meaningful effect sizes a factor in study design? A systematic review of translational chronic variable stress studies**

**Supporting Text**

*1. Key indicators of study quality*

In addition to documenting reporting and justification of sample sizes in our sample of articles using CVS, we also systematically documented other key indicators of study quality: use of exclusion criteria, use of random allocation to experimental groups, and experimenter blinding to groups (Landis et al., 2012). To do this, the online text of each article was searched using the terms “exclu”, “inclu”, “criteri”, “random” and “blind”.

*1.1 Exclusion criteria*

Seventy-four articles (19%) reported using exclusion criteria for their study. Twenty-three articles reported criteria applied prior to data collection (for example, screening animals for their “baseline” sucrose preference (Berrio et al., 2024) or behaviour in the open field test (Rosso et al., 2022)), and forty-nine articles reported criteria applied after data collection (for example, excluding animals that climbed their own tails in an effort to escape during the tail suspension test (Stukalin et al., 2020)). Two articles listed both types of exclusion criteria. In line with other systematic reviews on translational studies (Chitolina et al., 2023; Gallas-Lopes et al., 2023), we found that only 19% of articles reported exclusion criteria for their study (Supplementary Data 2). Twenty-five articles (6%) described excluding data points that were deemed to be outliers, usually defined as being more than two standard deviations away from the mean. We note here that data points with a value is distant from the mean should not normally be excluded. This is because deliberately reducing the variability in one’s data can disfavour the null hypothesis and increase the risk of false positives (André, 2022). We would argue that excluding data based on *a priori* expectations of what data ‘should’ look like may introduce bias; the world is full of unusual phenotypes that reflect biological reality, and removing unexpected (but otherwise reliable) data may distort understanding. If unusual data points violate a statistical test’s assumptions, other forms of analysis are often available (for example, bootstrapping (Pollet & van der Meij, 2017)).

*1.2 Randomisation*

178 articles (46%) reported that experimental units were allocated randomly to the control and CVS groups. This is higher than the median of 33% reported in other reviews (Baker et al., 2014; Berrio et al., 2024; Brent et al., 2021; Carneiro et al., 2018; Chitolina et al., 2023; Eastwood et al., 2015; Farrell et al., 2014; Gallas-Lopes et al., 2023; Hesen et al., 2017; Kilkenny et al., 2009; Moja et al., 2014; Rooke et al., 2011), but still low overall. A further seventeen articles (4%) reported random allocation to groups *after* the CVS procedure had taken place (Supplementary Data 2).

*1.3 Blinding*

133 articles (35%) reported that experimenters were blinded during the collection and/or analysis of data. This is somewhat higher than the median of 21% reported in other reviews (Baker et al., 2014; Bara & Joffe, 2014; Berrio et al., 2024; Brent et al., 2021; Carneiro et al., 2018; Chitolina et al., 2023; Codorniu et al., 2018; Eastwood et al., 2015; Faggion Jr et al., 2011; Farrell et al., 2014; Gallas-Lopes et al., 2023; Hesen et al., 2017; Kilkenny et al., 2009; Moja et al., 2014; Rooke et al., 2011). However, in twenty-one of those articles, experimenters were not blinded during at least one of the behavioural tests used to evaluate the effects of CVS. Four articles (1%) reported that experimenters were not blinded throughout. In the remaining 64% of articles, it was unclear whether blinding was used (Supplementary Data 2).

*References*

André, Q. (2022). Outlier exclusion procedures must be blind to the researcher’s hypothesis. *Journal of Experimental Psychology: General*, *151*(1), 213–223. https://doi.org/10.1037/xge0001069

Baker, D., Lidster, K., Sottomayor, A., & Amor, S. (2014). Two Years Later: Journals Are Not Yet Enforcing the ARRIVE Guidelines on Reporting Standards for Pre-Clinical Animal Studies. *PLOS Biology*, *12*(1), e1001756. https://doi.org/10.1371/journal.pbio.1001756

Bara, M., & Joffe, A. R. (2014). The ethical dimension in published animal research in critical care: The public face of science. *Critical Care*, *18*(1), R15. https://doi.org/10.1186/cc13694

Berrio, J. P., Hestehave, S., & Kalliokoski, O. (2024). Reliability of sucrose preference testing following short or no food and water deprivation-a Systematic Review and Meta-Analysis of rat models of chronic unpredictable stress. *Translational Psychiatry*, *14*(1), 39. https://doi.org/10.1038/s41398-024-02742-0

Brent, M. B., Brüel, A., & Thomsen, J. S. (2021). A Systematic Review of Animal Models of Disuse-Induced Bone Loss. *Calcified Tissue International*, *108*(5), 561–575. https://doi.org/10.1007/s00223-020-00799-9

Carneiro, C. F. D., Moulin, T. C., Macleod, M. R., & Amaral, O. B. (2018). Effect size and statistical power in the rodent fear conditioning literature – A systematic review. *PLOS ONE*, *13*(4), e0196258. https://doi.org/10.1371/journal.pone.0196258

Chitolina, R., Gallas-Lopes, M., Reis, C. G., Benvenutti, R., Stahlhofer-Buss, T., Calcagnotto, M. E., Herrmann, A. P., & Piato, A. (2023). Chemically-induced epileptic seizures in zebrafish: A systematic review. *Epilepsy Research*, *197*, 107236. https://doi.org/10.1016/j.eplepsyres.2023.107236

Codorniu, A., Lemasle, L., Legrand, M., Blet, A., Mebazaa, A., & Gayat, E. (2018). Methods used to assess the performance of biomarkers for the diagnosis of acute kidney injury: A systematic review and meta-analysis. *Biomarkers*, *23*(8), 766–772. https://doi.org/10.1080/1354750X.2018.1493616

Eastwood, M. P., Russo, F. M., Toelen, J., & Deprest, J. (2015). Medical interventions to reverse pulmonary hypoplasia in the animal model of congenital diaphragmatic hernia: A systematic review. *Pediatric Pulmonology*, *50*(8), 820–838. https://doi.org/10.1002/ppul.23206

Faggion Jr, C. M., Giannakopoulos, N. N., & Listl, S. (2011). Risk of bias of animal studies on regenerative procedures for periodontal and peri-implant bone defects – a systematic review. *Journal of Clinical Periodontology*, *38*(12), 1154–1160. https://doi.org/10.1111/j.1600-051X.2011.01783.x

Farrell, K. E., Keely, S., A. Graham, B., Callister, R., & Callister, R. J. (2014). A Systematic Review of the Evidence for Central Nervous System Plasticity in Animal Models of Inflammatory-mediated Gastrointestinal Pain. *Inflammatory Bowel Diseases*, *20*(1), 176–195. https://doi.org/10.1097/01.MIB.0000437499.52922.b1

Gallas-Lopes, M., Bastos, L. M., Benvenutti, R., Panzenhagen, A. C., Piato, A., & Herrmann, A. P. (2023). Systematic review and meta-analysis of 10 years of unpredictable chronic stress in zebrafish. *Lab Animal*, *52*(10), Article 10. https://doi.org/10.1038/s41684-023-01239-5

Hesen, N. A., Riksen, N. P., Aalders, B., Ritskes-Hoitinga, M., Messaoudi, S. E., & Wever, K. E. (2017). A systematic review and meta-analysis of the protective effects of metformin in experimental myocardial infarction. *PLOS ONE*, *12*(8), e0183664. https://doi.org/10.1371/journal.pone.0183664

Kilkenny, C., Parsons, N., Kadyszewski, E., Festing, M. F. W., Cuthill, I. C., Fry, D., Hutton, J., & Altman, D. G. (2009). Survey of the Quality of Experimental Design, Statistical Analysis and Reporting of Research Using Animals. *PLOS ONE*, *4*(11), e7824. https://doi.org/10.1371/journal.pone.0007824

Landis, S. C., Amara, S. G., Asadullah, K., Austin, C. P., Blumenstein, R., Bradley, E. W., Crystal, R. G., Darnell, R. B., Ferrante, R. J., Fillit, H., Finkelstein, R., Fisher, M., Gendelman, H. E., Golub, R. M., Goudreau, J. L., Gross, R. A., Gubitz, A. K., Hesterlee, S. E., Howells, D. W., … Silberberg, S. D. (2012). A call for transparent reporting to optimize the predictive value of preclinical research. *Nature*, *490*(7419), Article 7419. https://doi.org/10.1038/nature11556

Moja, L., Pecoraro, V., Ciccolallo, L., Dall’Olmo, L., Virgili, G., & Garattini, S. (2014). Flaws in animal studies exploring statins and impact on meta-analysis. *European Journal of Clinical Investigation*, *44*(6), 597–612. https://doi.org/10.1111/eci.12264

Pollet, T. V., & van der Meij, L. (2017). To Remove or not to Remove: The Impact of Outlier Handling on Significance Testing in Testosterone Data. *Adaptive Human Behavior and Physiology*, *3*(1), 43–60. https://doi.org/10.1007/s40750-016-0050-z

Rooke, E. D. M., Vesterinen, H. M., Sena, E. S., Egan, Kieren. J., & Macleod, M. R. (2011). Dopamine agonists in animal models of Parkinson’s disease: A systematic review and meta-analysis. *Parkinsonism & Related Disorders*, *17*(5), 313–320. https://doi.org/10.1016/j.parkreldis.2011.02.010

Rosso, M., Wirz, R., Loretan, A. V., Sutter, N. A., Pereira da Cunha, C. T., Jaric, I., Würbel, H., & Voelkl, B. (2022). Reliability of common mouse behavioural tests of anxiety: A systematic review and meta-analysis on the effects of anxiolytics. *Neuroscience & Biobehavioral Reviews*, *143*, 104928. https://doi.org/10.1016/j.neubiorev.2022.104928

Stukalin, Y., Lan, A., & Einat, H. (2020). Revisiting the validity of the mouse tail suspension test: Systematic review and meta-analysis of the effects of prototypic antidepressants. *Neuroscience & Biobehavioral Reviews*, *112*, 39–47. https://doi.org/10.1016/j.neubiorev.2020.01.034
